# Osteoprotegerin and Myocardial Fibrosis in Patients with Aortic Stenosis

**DOI:** 10.1038/s41598-018-32738-y

**Published:** 2018-09-28

**Authors:** Brodie L. Loudon, Eleana Ntatsaki, Simon Newsome, Brian Halliday, Amrit Lota, Aamir Ali, Tamir Malley, Subothini Selvendran, Nikhil Aggarwal, Willis Lam, Jackie Donovan, Dominque Auger, Claire E. Raphael, Paul D. Flynn, Dudley J. Pennell, Vassilios S. Vassiliou, Sanjay K. Prasad

**Affiliations:** 1 CMR Unit, Royal Brompton Hospital and NIHR Biomedical Research Unit, Royal Brompton and Harefield Hospitals, London, United Kingdom; 2 Norwich Medical School, University of East Anglia, Bob Champion Research & Education Building and Norfolk and Norwich University Hospital, Norwich, United Kingdom; 3 Department of Rheumatology, Ipswich Hospital NHS Trust, and University College London, Ipswich, United Kingdom; 4 Imperial College London, London, United Kingdom; 5 Department of Biochemistry, Royal Brompton Hospital, London, United Kingdom; 6 Department of Metabolic Medicine, Addenbrooke’s Hospital, Cambridge and University of Cambridge, Cambridge, United Kingdom

## Abstract

Left ventricular myocardial fibrosis in patients with aortic stenosis (AS) confers worse prognosis. Plasma osteoprotegerin (OPG), a cytokine from the TNF receptor family, correlates with the degree of valve calcification in AS, reflecting the activity of the tissue RANKL/RANK/OPG (receptor activator of nuclear factor κΒ ligand/RANK/osteoprotegerin) axis, and is associated with poorer outcomes in AS. Its association with myocardial fibrosis is unknown. We hypothesised that OPG levels would reflect the extent of myocardial fibrosis in AS. We included 110 consecutive patients with AS who had undergone late-gadolinium contrast enhanced cardiovascular magnetic resonance (LGE-CMR). Patients were characterised according to pattern of fibrosis (no fibrosis, midwall fibrosis, or chronic myocardial infarction fibrosis). Serum OPG was measured with ELISA and compared between groups defined by valve stenosis severity. Some 36 patients had no fibrosis, 38 had midwall fibrosis, and 36 had chronic infarction. Patients with midwall fibrosis did not have higher levels of OPG compared to those without fibrosis (6.78 vs. 5.25 pmol/L, p = 0.12). There was no difference between those with midwall or chronic myocardial infarction fibrosis (6.78 vs. 6.97 pmol/L, p = 0.27). However, OPG levels in patients with chronic myocardial infarction fibrosis were significantly higher than those without fibrosis (p = 0.005).

## Introduction

Calcification of the cardiovascular system occurs commonly with aging. Progressive aortic valve calcification occurs in some patients, with symptoms presenting once critical restriction of valve-opening and left ventricular pressure overload ensue. The mechanisms responsible for the pathogenesis of calcific aortic stenosis (AS) are unknown, and likely reflect the interplay of multiple cytokine pathways and genetic factors^1,2^. Determining the ideal timing for aortic valve replacement (AVR) in AS remains a challenge. Current guideline-based indications for intervention depend on the severity of valve narrowing, as usually assessed with echocardiography, the presence of symptoms relating to a narrowed valve, and impairment of systolic function, as assessed by left ventricular ejection fraction (LVEF)^3,4^. Unfortunately, stenosis severity on echocardiography does not correlate well with the risk of progression of LV hypertrophy to heart failure, which is primarily driven by the development of myocardial fibrosis^5^. Currently, cardiovascular magnetic resonance (CMR) remains the only non-invasive, radiation-free investigation enabling the visualisation of myocardial fibrosis in the late phase following gadolinium administration indicating myocardial scar^6^. However, the associated costs and limited access to CMR limit its routine use in all patients with AS. It is also recognised that patients with fibrosis have worse prognosis independently of LVEF^7^, and therefore formulating an easy, non-invasive, and cost-effective way of identifying patients with high prevalence of fibrosis would be clinically useful. Blood biomarkers have the potential to identify patients with fibrosis and lend themselves to serial monitoring of these patients. In this way, they may help to identify those patients in whom greatest benefit may be gained from early AVR. Recently, the RANKL/RANK/OPG (receptor activator of nuclear factor κΒ ligand/RANK/osteoprotegerin) system, known to play an important role in bone turnover and vascular calcification^8^, has gained much interest in AS^9^. Osteoprotegerin (OPG) is a member of the tumour necrosis factor (TNF) receptor family and inhibits the interaction between RANKL and RANK^10,11^, and therefore may represent one such biomarker.

Most studies of tissue levels of OPG within the diseased aortic valve report a reduction in tissue OPG compared to controls^9,12,13^, but increased mRNA levels^13^. In contrast, reported levels of serum OPG consistently show a rise in tandem with an increase in tissue RANKL/RANK activity (but not serum RANKL^14^), in patients with a range of cardiovascular conditions, including vascular calcification^15–17^, diabetes^18^, and heart failure^19–21^. The same has also been demonstrated in AS^22–24^. Recently, preoperative serum OPG was shown to correlate with a worse outcome after AVR in 124 patients with severe AS during a mean follow-up of 3.8 years^23^. Additionally in other studies^22–24^, OPG levels were associated with AS severity, pulmonary capillary wedge pressure, and NT-proBNP levels, indicating that the presence of HF may be an important determinant of serum OPG in AS. Interestingly, OPG levels also correlated with LV global longitudinal strain (GLS) and strain rate, but ejection fraction (EF) did not differ between OPG tertiles^23^. Subtle changes in LV longitudinal function often precede changes in EF^25–27^, and elevated OPG levels may therefore suggest underlying myocardial fibrosis, which drives the progression to HF^5^. Patients with severe AS and cardiovascular magnetic resonance (CMR)-derived measures of myocardial replacement fibrosis (either midwall or chronic myocardial infarction) have poorer outcomes^28,29^. This has also been confirmed in a large prospective observational cohort study of patients with less severe disease^30^. Inflammation is known to play a critical role in the development of LV myocardial fibrosis^31^, and can also activate the RANKL/RANK/OPG system^32^. In an isoproterenol-induced rat model of heart failure, RANKL/RANK were shown to be crucial mediators of interleukin-17 (IL-17) induced activation of matrix metalloproteinase-1 (MMP-1) in cardiac fibroblasts^33^. In this model, treatment with both OPG and inhibitors of IL-17 reduced myocardial fibrosis.

The association between OPG levels and myocardial replacement fibrosis in AS remains unknown. The main aim of our study was to determine whether serum OPG was associated with the presence of (1) myocardial midwall and (2) myocardial infarction fibrosis in patients with AS, and compare patients with mild/moderate and severe disease. Additionally, we investigated the prognostic role of OPG in patients with AS.

## Methods

Consecutive patients with AS and late gadolinium contrast enhanced CMR (LGE-CMR) were included in this prospective substudy^34^ (ClinicalTrials.gov Identifier: NCT00930735). AS severity was assessed according to the most recent AHA/ACC clinical practice guidelines for management of patients with valvular heart disease^3^. Exclusion criteria included acute coronary syndrome, clinical suspicion or evidence of infection, disseminated malignancy, severe aortic regurgitation, more than moderate mitral regurgitation/stenosis, previous valve replacement, contraindication to CMR (including presence of non-conditional devices), and estimated glomerular filtration rate of <30 ml/min. This study was approved by the NHS England Research Ethics Committee, and was undertaken in line with the ethical standards of the Declaration of Helsinki. All patients provided written informed consent.

### Cardiovascular Magnetic Resonance

CMR scans were performed on a 1.5 T scanner (Magnetom Sonata or Avanto, Siemens, Erlangen, Germany) using a standardised protocol as previously described^34^. In short, initial localiser images were used to guide acquisition of a vertical long axis (VLA) cine with balanced steady state free precession (SSFP) at end-expiration. This was then used to guide SSFP cines in the two-, three- and four-chamber views. Contiguous 10 mm short axis slices of the LV were then taken from base to apex. Retrospective ECG gating was preferred for the cine acquisition. In patients with arrhythmia, prospective triggering was utilised. Aortic valve planimetry and LV mass and volume were then calculated. After gadolinium contrast agent (Gadovist, Schering AG, Berlin, Germany) administration, inversion recovery-prepared spoiled gradient echo images were acquired in standard long- and short-axis views to detect areas of LGE^28^. Images were analysed offline on dedicated software for LV function, volumes, mass, and AS severity (*CMR Tools*, Cardiovascular Imaging Solutions., London, United Kingdom). LV myocardial fibrosis was quantified via separate dedicated software (*CVI*^42^, Circle Cardiovascular Imaging, Calgary, Canada). Two independent, blinded, expert observers analysed the images with a third blinded observer adjudicating in cases of disagreement.

### Osteoprotegerin Levels

OPG measurement from stored plasma was undertaken using monoclonal anti-human OPG antibody. Detection was via biotin-labelled polyclonal anti-human OPG antibody and Streptavidin HRP conjugate (*BioVendor, Research and Diagnostics Products)*. This produced a yellow colour whose absorbance was measured at 450 nm. A reference absorbance of 620 nm was also measured and subtracted from all samples before calculating concentrations. The absorbance was proportional to the concentration of OPG in the sample, allowing detection of OPG from 3–60 pmol/L.

### Statistical analysis

Baseline data are presented as mean ± SD for continuous variables and number (proportion) for categorical variables. Mann-Whitney U tests were used to test for either an association between OPG levels and AS severity, or OPG levels and presence (or absence) of myocardial fibrosis. Univariable and multivariable linear regression analyses were performed to identify possible predictors of OPG levels. A p value of <0.05 was taken as significant. All analyses were undertaken using Stata version 14.0 (College Station, Texas, USA).

## Results

Patient data are shown in Table 1. Mild/moderate AS was diagnosed in 35 (31.8%) patients, and severe AS in 75 (68.2%) patients. Compared to patients with mild/moderate disease, patients with severe disease were older (78 ± 9 years vs 71 ± 10, p < 0.001), but well-matched in terms of gender, ethnicity, body mass index (BMI), cardiovascular comorbidities and resting blood pressure. Patients with severe disease also had more severe symptoms by New York Heart Association (NYHA) symptom class (≥class II, 60 (81.1%) vs 19 (59.4%), p = 0.03). Drug therapy was very similar between groups, with the exception of calcium channel blocker therapy being more common in the patient group with mild/moderate disease (8/26.7%) compared to patients with severe disease (6/8.7%, p = 0.03). NT-proBNP levels were higher in patients with severe AS compared to those with mild/moderate disease (1232 pg/ml vs. 3813 pg/ml; p = 0.002); however serum CRP levels did not differ between the groups (12 mg/L vs. 15 mg/L; p = 0.52). Serum creatinine was also similar between mild/moderate and severe groups (93 μmol/L vs 102 μmol/L, p = 0.20). The correlations between demographic, biochemical, and CMR data with serum OPG are presented in Table 2. Age (p < 0.0001), weight (p < 0.0001), serum creatinine (p = 0.001), diuretic use (p = 0.009), serum NT-proBNP (p = 0.002), CRP (p = 0.038), valve area on CMR (p = 0.011), LV mass (p = 0.027), and LGE % (p = 0.01) correlated with serum OPG. Baseline patient data were also well-matched across fibrosis-pattern groups (Supplementary Data Table 1).

**Table 1:** Baseline Patient Data.

Demographics	Mild/Moderate (N = 35)	Severe (N = 75)	p Value
Age, years	71 ± 10	78 ± 9	<0.001*
Male, n (%)	26 (74.3)	51 (68.0)	0.66
Hypertension, n (%)	13 (40.6)	44 (58.7)	0.096
SBP, mmHg	130 ± 19	130 ± 22	0.95
DBP, mmHg	71 ± 11	71 ± 13	0.46
Diabetes mellitus, n (%)	1 (3.8)	4 (6.1)	1.00
Current smoker, n (%)	2 (6.3)	3 (4.1)	0.64
Any coronary artery disease, n (%)	13 (37.1)	27 (36.0)	1.00
Previous stroke, n (%)	1 (2.9)	2 (2.7)	1.00
Atrial Fibrillation, n (%)	5 (14.3)	6 (8.0)	0.32
Hypercholesterolaemia, n (%)	18 (58.1)	50 (67.6)	0.38
NYHA Class ≥ II	19 (59.4)	60 (81.1)	0.03*
Caucasian	33 (94.3)	72 (96.0)	0.65
Height, cm	172 ± 12	168 ± 10	0.03*
Weight, kg	82 ± 18	75 ± 17	0.05
BMI, kg/m^2^	28 ± 5	26 ± 5	0.17
**Pharmacotherapy**			
Aspirin, n (%)	19 (61.3)	44 (59.5)	1.00
Clopidogrel, n (%)	4 (13.8)	12 (16.7)	1.00
ACE-I/ARB, n (%)	13 (43.3)	38 (51.4)	0.52
Beta Blocker, n (%)	14 (46.7)	32 (44.4)	1.00
Calcium channel blocker, n (%)	8 (26.7)	6 (8.7)	0.03*
Diuretic, n (%)	14 (43.8)	43 (58.1)	0.21
Warfarin, n (%)	4 (14.3)	6 (8.3)	0.46
Amiodarone, n (%)	0 (0.0)	4 (5.6)	0.32
Statin, n (%)	20 (66.7)	54 (72.0)	0.64
**Biochemical data**			
Osteoprotegerin, pmol/L	5.76 ± 1.96	7.91 ± 3.82	0.002*
Creatinine, μmol/L	93 ± 30	102 ± 36	0.20
NT-ProBNP (pg/mL)	1232 ± 1698	3813 ± 5489	0.002*
CRP (mg/L)	12 ± 22	15 ± 39	0.52
**CMR data**			
CMR aortic valve area, cm^2^	1.2 ± 0.3	0.7 ± 0.1	<0.00001*
LVEF, %	62 ± 14	57 ± 17	0.11
LV Mass, g	166 ± 47	168 ± 57	0.87
No Myocardial Fibrosis, n (%)	13 (37.1)	23 (30.7)	0.82
Midwall Fibrosis, n (%)	11 (31.4)	27 (36.0)	0.82
Infarction Pattern Fibrosis, n (%)	11 (31.4)	25 (33.3)	0.82
LGE Mass	5.7 (7.6)	6.0 (7.9)	0.72
LGE Percent	3.4 (4.8)	3.8 (5.1)	0.71
**Intervention**			
None	21 (60.0)	24 (32.0)	0.01*
AVR	9 (25.7)	25 (33.3)	0.01*
TAVR	5 (14.3)	26 (34.7)	0.01*

Values are mean ± SD unless otherwise stated. *p < 0.05. Baseline data for AS patients based on severity. ACE-I, angiotensin converting enzyme inhibitor; ARB, angiotensin II receptor blocker; AVR, aortic valve replacement; BMI, body mass index; CMR, cardiovascular magnetic resonance; DBP, diastolic blood pressure; LGE, late gadolinium enhancement; LVEF, left ventricular ejection fraction; NYHA, New York Heart Association; SBP, systolic blood pressure; TAVR, transcatheter aortic valve replacement.

**Table 2:** Correlations between Patient Data and Serum OPG levels.

Variable	Spearman’s Correlation with OPG	P-Value
**Patient Demographics**		
Age, years	0.56	<0.0001*
Male, n (%)	−0.025	0.80
Hypertension, n (%)	−0.047	0.63
SBP, mmHg	0.015	0.88
DBP, mmHg	−0.13	0.19
Diabetes mellitus, n (%)	0.024	0.82
Current smoker, n (%)	0.005	0.96
Any coronary artery disease, n (%)	0.10	0.29
Previous stroke, n (%)	0.0009	0.99
Atrial Fibrillation, n (%)	−0.023	0.82
Hypercholesterolaemia, n (%)	0.028	0.78
NYHA ≥ II	0.033	0.73
Height, cm	−0.17	0.077
Weight, kg	−0.37	<0.0001*
BMI, kg/m^2^	−0.36	<0.001*
**Pharmacotherapy**		
Aspirin, n (%)	0.006	0.95
Clopidogrel, n (%)	0.046	0.65
ACE-I/ARB, n (%)	−0.11	0.26
Beta Blocker, n (%)	−0.098	0.33
Calcium channel blocker, n (%)	−0.25	0.014*
Diuretic, n (%)	0.25	0.009*
Warfarin, n (%)	0.061	0.55
Amiodarone, n (%)	−0.071	0.48
Statin, n (%)	−0.046	0.64
**Biochemical Data**		
Creatinine, μmol/L	0.32	0.001*
NT-ProBNP (pg/mL)	0.30	0.002*
CRP (mg/L)	0.22	0.038*
**CMR Data**		
CMR aortic valve area, cm^2^	−0.24	0.011*
LVEF, %	−0.041	0.67
LV Mass, g	−0.22	0.021*
LGE Mass	0.22	0.027*
LGE Percent	0.25	0.010*

*p < 0.05. Correlation between Patient Data and Serum OPG Levels. ACE-I, angiotensin converting enzyme inhibitor; ARB, angiotensin II receptor blocker; BMI, body mass index; CMR, cardiovascular magnetic resonance; DBP, diastolic blood pressure; LGE, late gadolinium enhancement; LVEF, left ventricular ejection fraction; NYHA, New York Heart Association; SBP, systolic blood pressure.

### CMR Data

By definition, aortic valve orifice area on CMR was significantly reduced in the severe group compared to the mild/moderate group (0.7 ± 0.1 cm^2^ vs 1.2 ± 0.3 cm^2^, p < 0.00001). Ejection fraction and LV mass did not differ between these groups. Midwall fibrosis was detected in 11 (31.4%) patients with mild/moderate disease and 27 (36.0%) patients with severe disease (mild/moderate vs severe, p = 0.82). Chronic infarction pattern fibrosis was detected in 11 (31.4%) patients with mild/moderate disease and 25 (33.3%) patients with severe disease (mild/moderate vs severe, p = 0.82). Myocardial fibrosis was absent in 13 (37.1%) patients with mild/moderate disease and 23 (30.7%) patients with severe disease (mild/moderate vs severe, p = 0.82).

### Surgical Intervention

Significantly fewer patients with mild/moderate disease underwent an intervention compared to patients with severe disease (14 patients vs 51 patients, 40% vs 68%, p = 0.01). Some 9/14 (64.3%) patients with moderate disease underwent aortic valve replacement surgery (AVR) concurrently with coronary artery bypass grafting (CABG) surgery for ischaemic heart disease, compared to 25/51 (49.0%) patients with severe disease (mild/moderate vs severe, p = 0.01).

### Aortic Stenosis Severity and OPG Levels

**Figure 1:**
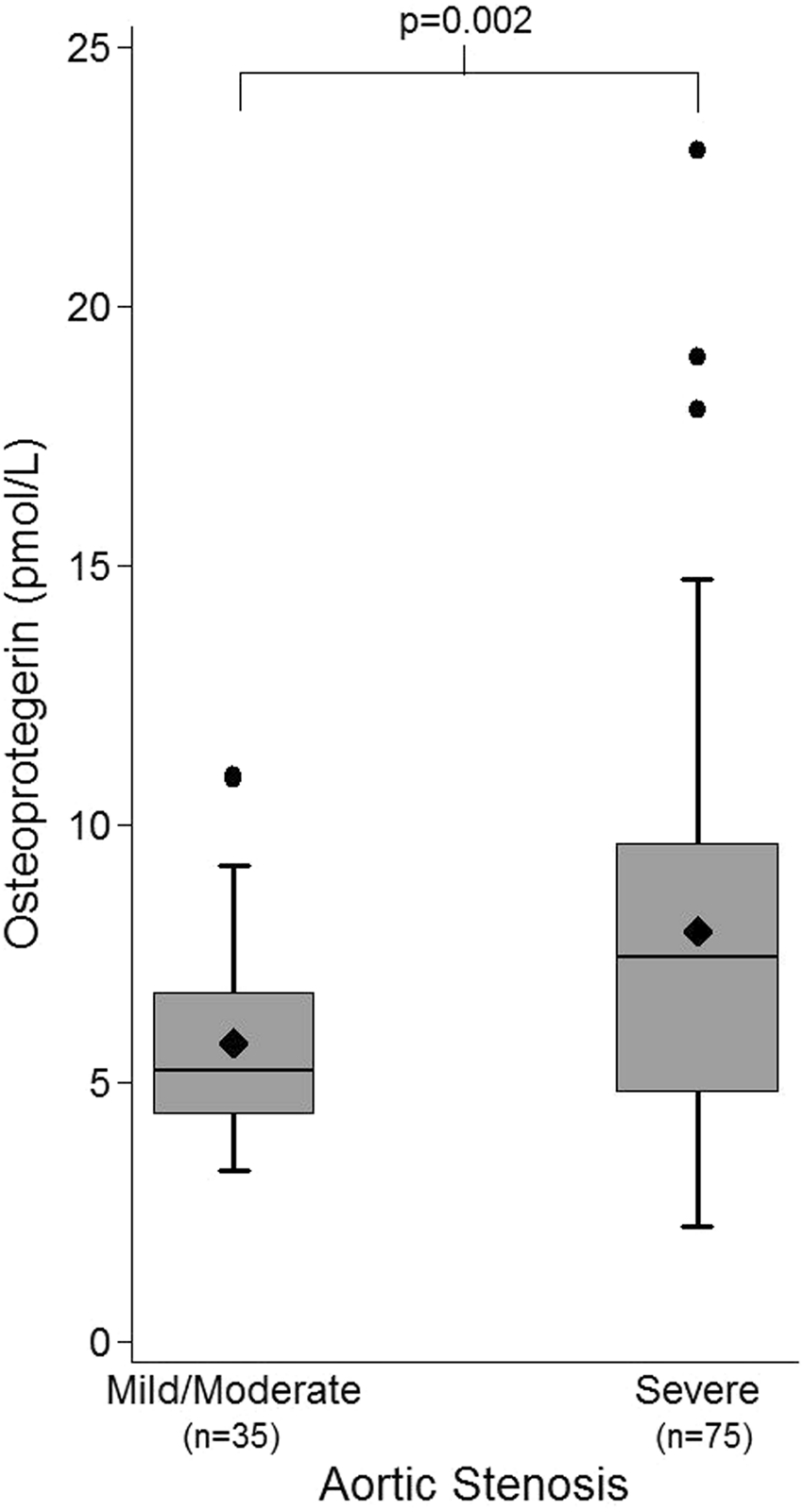
Box and Whisker Plots of Osteoprotegerin levels by Aortic Stenosis Severity. Patients with severe disease had higher serum OPG levels than those with mild/moderate disease (7.91 ± 3.82 pmol/L vs 5.76 ± 1.96 pmol/L, p = 0.002). Diamond represents the mean OPG level. Outlier data are included on the plots.

Serum OPG levels in patients with mild/moderate disease were a median of 5.24 pmol/L (IQR 4.38–6.76 pmol/L). In patients with severe disease, OPG levels were a median of 7.43 pmol/L (IQR 4.80–9.63 pmol/L), which was significant greater than the levels in patients with mild/moderate disease (p = 0.002, Fig. 1). CMR valve orifice area correlated with serum OPG levels (r_s_ = −0.24, p = 0.011; Table 2). Following linear regression analysis, the severity of aortic stenosis had a significant effect on OPG levels (p = 0.0002). On multivariable analysis (adjusting for age, sex, presence of fibrosis, LVEF, NYHA, NT-proBNP, and CRP) severe AS remained significant, increasing serum OPG by 1.30 pmol/L (95% CI 0.002–2.60 pmol/L, p = 0.049). A further multivariable analysis was performed to exclude the effect of concomitant cardiovascular disease in patients with severe AS on serum OPG levels. Adjusting for CAD, diabetes, and hypertension, AS severity was an independent predictor of serum OPG (p = 0.008).

In order to investigate whether serum OPG levels could predict severe AS in patients, receiver operator curves were constructed, and the area under the ROC curve (AUC) was calculated. The AUC for AS severity was 0.69 (Supplementary Data, Fig. 1).

### Low-Gradient Aortic Stenosis

Low-gradient AS, defined as a stroke volume (SV) index <35 ml/m^2^, was identified in 16 (14.5%) patients. Of these, 3 had mild/moderate AS, and the remaining 13 had severe AS. There was no difference in AS severity between patients with low-gradient AS and those without (p = 0.38). CMR identified no LV fibrosis in 7 patients, midwall pattern fibrosis in 6 patients, and chronic infarction pattern fibrosis in 3 patients. Serum OPG levels were similar between patients with low-gradient AS and those without (7.0 ± 2.9 pmol/L vs. 7.3 ± 3.6 pmol/L; p = 0.90).

### Effect of Myocardial Fibrosis on OPG Levels

**Figure 2:**
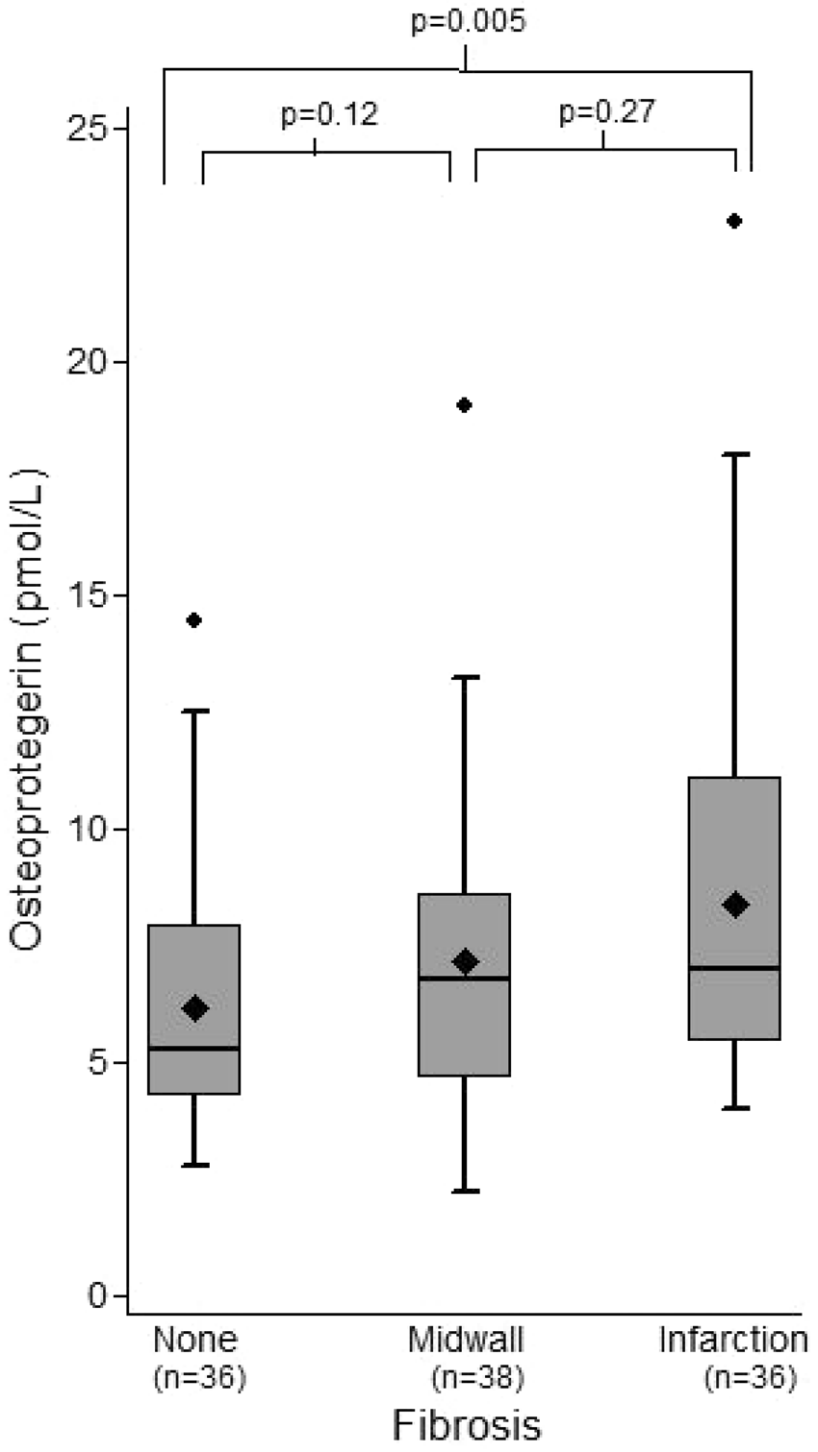
Box and Whisker Plots of Osteoprotegerin levels by pattern of Myocardial Fibrosis on CMR. Patients with midwall fibrosis on CMR did not have significantly higher levels of OPG compared to those without fibrosis (median 6.78 pmol/L [IQR 4.64–8.61] vs median 5.25 pmol/L [IQR 4.26–7.95 pmol/L], p = 0.12). There was no difference between those with midwall or chronic infarction pattern fibrosis (median 6.78 pmol/L [IQR 4.64–8.61 pmol/L] vs median 6.97 pmol/L [IQR 5.43–11.12 pmol/L], p = 0.27). However, OPG levels in patients with chronic infarction fibrosis were significantly higher than those without fibrosis (p = 0.005). Diamond represents the mean OPG level. Outlier data are included on the plots.

LGE % on CMR correlated with serum OPG (r_s_ = 0.25, p = 0.01; Table 2). The median serum OPG for patients without myocardial fibrosis on CMR (n = 36) was 5.25 pmol/L (IQR 4.26–7.95 pmol/L). Patients with midwall fibrosis (n = 38) had a median serum OPG of 6.78 pmol/L (IQR 4.64–8.61 pmol/L), which was not significantly higher than those with no fibrosis (p = 0.12). Patients with a chronic infarction pattern of fibrosis had a median serum OPG of 6.97 pmol/L (IQR 5.43–11.12 pmol/L), which was significantly higher than those without fibrosis (p = 0.005) but not higher than those with midwall fibrosis (p = 0.27, Fig. 2). On linear regression analysis, ‘any fibrosis’ (midwall or infarction) had a significant effect on OPG levels (p = 0.01). On multivariable analysis (adjusting for age, sex, AS severity, & LVEF) however, a significant effect was no longer observed (p = 0.20). There was also no significant correlation between fibrosis and OPG levels when adjusting for CAD, diabetes, and hypertension (p = 0.11). Separating out midwall and chronic infarction patterns of fibrosis, linear regression analysis showed a significant effect of chronic infarction pattern on OPG levels (HR 2.21, 95% CI 0.57, 3.86; p = 0.009), but not of midwall fibrosis pattern (HR 1.00, 95% CI −0.36, 2.36; p = 0.15).

**Figure 3:**
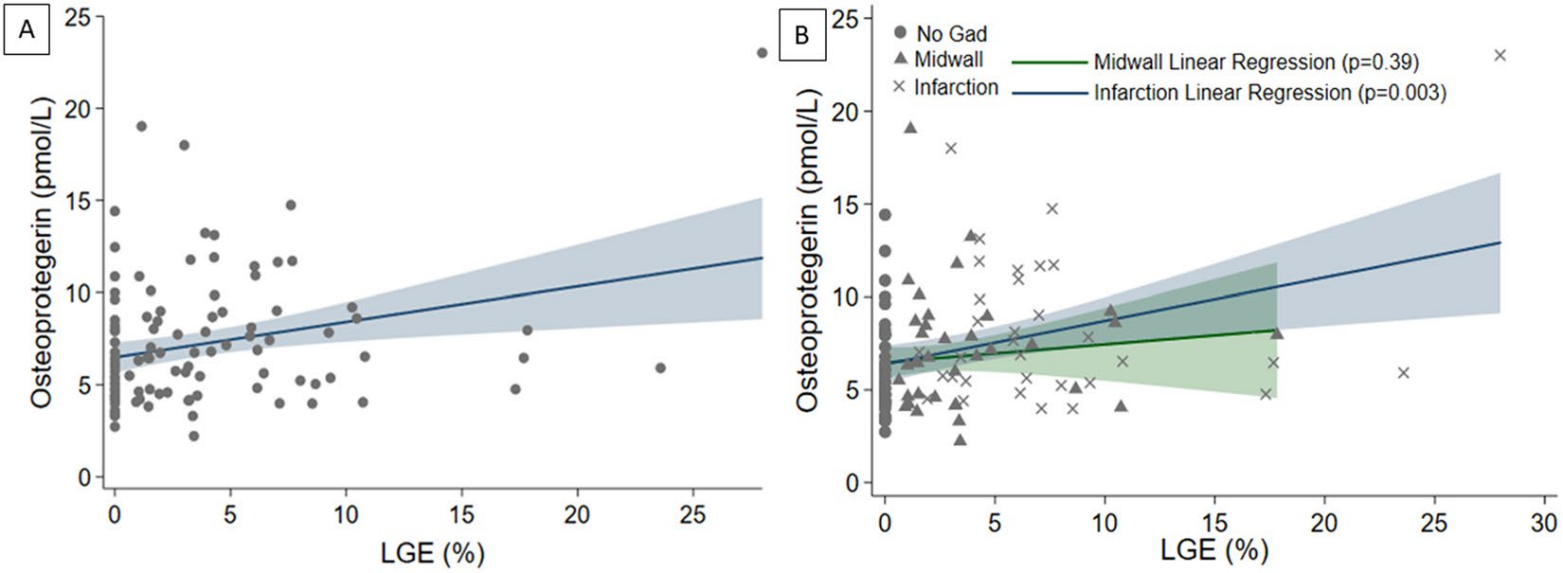
Effect of Extent of Fibrosis on OPG per %LGE on CMR. Panel (A) On multivariable linear regression analysis, ‘any’ fibrosis (per % increase in LGE on CMR) increased serum OPG by 0.16 pmol/L (95% CI 0.03–0.30 pmol/L, p = 0.02). Panel (B) Separating out patients based on pattern of fibrosis, midwall fibrosis had no effect on OPG levels (0.10 pmol/L, 95% CI −0.13–0.32, p = 0.39), whereas chronic infarction pattern fibrosis increased serum OPG by 0.19 pmol/L (95% CI 0.03–0.35 pmol/L) for every 1% increase in LGE on CMR (p = 0.02).

The effect of ‘any fibrosis’ on serum OPG per 1% increase in LGE on CMR was significant on univariable linear regression analysis (p = 0.005). On multivariable analysis, this remained significant, increasing serum OPG by 0.16 pmol/L (95% CI 0.03–0.30 pmol/L) per 1% increase in LGE (p = 0.02, Fig. 3A). Separating out patterns of fibrosis on CMR, there was no significant effect of midwall fibrosis on linear regression on serum OPG (p = 0.15) and this remained true on multivariable analysis (p = 0.19). However, there was a significant effect of chronic myocardial infarction pattern fibrosis univariable analysis (p = 0.009; Fig. 3B). On multivariable analysis, however, this effect only showed a strong trend (HR 2.11, 95% CI −0.10, 4.32; p = 0.061). Age (p = 0.004), LVEF (p = 0.035), and CRP (p = 0.017) each remained significant on the multivariable analysis.

Serum OPG levels predicted LV myocardial fibrosis with an AUC of 0.65 (Supplementary Data, Fig. 2).

### Left Ventricular Ejection Fraction and Serum OPG Levels

**Figure 4:**
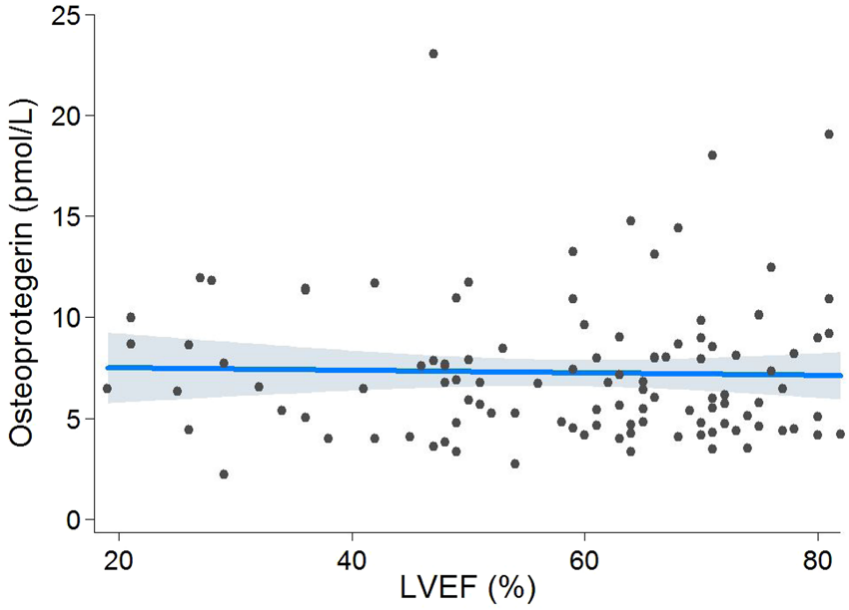
Effect of EF on OPG Levels. On multivariable linear regression analysis (adjusted for age, sex, AS severity, and presence of myocardial fibrosis), serum OPG levels remained unchanged per 10% change in LV ejection fraction (0.18 pmol/L, 95% CI −0.23–0.59 pmol/L, p = 0.39).

LV ejection fraction did not correlate with serum OPG levels (r_s_ = −0.04, p = 0.67; Table 2). CMR-derived ejection fraction was not significantly different between patients with mild/moderate disease and those with severe disease (62 ± 14% vs 57 ± 17%, p = 0.11). Per 10% change, LV ejection fraction did not have a significant effect on serum OPG (p = 0.77). On multivariable analysis (adjusted for age, sex, AS severity, and presence of myocardial fibrosis), there was still no effect observed (p = 0.39, Fig. 4).

### NT-proBNP and Serum OPG Levels

NT-proBNP correlated with serum OPG levels in patients (r_s_ = 0.30, p = 0.002; Table 2). A total of 64 (58.2%) patients met the diagnostic criteria for the heart failure with preserved ejection fraction (HFpEF) syndrome, characterised by heart failure symptoms, LV EF ≥ 50%, and a serum NT-proBNP > 125 pg/ml^36^. Serum OPG levels did not differ significantly between patients with HFpEF and those without (7.1 ± 3.6 pmol/L vs. 7.3 ± 3.5 pmol/L; p = 0.74). Patients with HFpEF were not more likely to have severe AS (p = 0.49), nor either pattern of fibrosis on CMR (p = 0.31).

### Effect of OPG on survival

There were 30 (27.3%) deaths over the median follow-up period of 1.9 years (IQR 1.2–2.7 years). Using the Cox proportional hazards method, serum OPG had a significant effect on survival on univariable analysis (p < 0.0001). On multivariable analysis (adjusted for age, sex, AVR/TAVR intervention, presence of myocardial fibrosis, NYHA class, CAD, diabetes, and hypertension), this effect remained statistically significant, with every 1 pmol/L increase in baseline serum OPG increasing the risk of death by 19% (HR 1.19, 95% CI 1.06–1.34; p = 0.004).

## Discussion

This is the first study to investigate the association between myocardial fibrosis on CMR and serum OPG levels in patients with AS. There was no association between midwall fibrosis and serum OPG levels in our study. However, for patients with chronic infarction pattern fibrosis on CMR, there was an effect on serum OPG on univariable analysis (p = 0.009), but there was only a trend on multivariable analysis when accounting for age, sex, AS severity, LVEF, NYHA, NT-proBNP, and CRP (p = 0.061). We included patients with both mild/moderate and severe AS, and confirmed the findings of others that disease severity is independently associated with raised serum OPG^22–24^. The patient groups were also well-matched in terms of baseline characteristics and medical therapy. Specifically, statin therapy, which reduces inflammatory (TNFα mediated) OPG release by endothelial cells^37^, did not differ between groups. While LVEF did not appear to have a significant effect on serum OPG in the current study, only 15 patients (13.6%) had a LVEF ≤ 40%, complicating the assessment of the effect of concomitant heart failure on OPG in our patient cohort. On multivariable analysis, the raised serum OPG in patients with chronic ischaemic pattern fibrosis on CMR appeared to associate more with older age and higher levels of serum NT-proBNP and CRP. Importantly, we have also confirmed the association between higher serum OPG and poorer mid-term survival (median 1.9 years in the present study) in patients with AS^23^, demonstrating a 19% increase in the risk of death per 1 pmol/L rise in serum in OPG. This is clinically important, as it appears that the risk associated with OPG elevation is independent of LVEF and midwall myocardial fibrosis.

In addition to regulating bone calcification^38^, the RANKL/RANK/OPG pathway also plays a key role in immune function via modulation of dendritic cell function, and is upregulated in T lymphocytes during inflammation^39^. In addition to blockade of RANK, OPG also interacts with tumour necrosis factor related apoptosis inducing ligand (TRAIL), to block TRAIL-related apoptosis^40^. The RANKL/RANK/OPG axis is also active within the vasculature and has been shown to play a major role in pathological vascular calcification, in a manner analogous to bone turnover^8^, and is upregulated in accelerated atherosclerotic plaques^16^. OPG has been shown to induce vascular fibrosis in aortic samples of apolipoprotein E knockout (ApoE[−/−]) mice, via transforming growth factor-β1 (TGF-β1), which also mediates OPG release in response to angiotensin II in vascular smooth muscle cells^41^. A recent meta-analysis including a total of 26,442 participants from the general population demonstrated an association between raised baseline serum OPG and an increased risk of incident cardiovascular disease at a mean follow-up of 8.5 years, independent of age and clinically-relevant risk factors^35^.

Defining the role of OPG in patients with AS may therefore be complicated by the presence of concomitant cardiovascular disease. There also appears to be a difference between the actions of OPG in vascular and cardiac tissue in mouse models, as it has shown to be pro-fibrotic in the former^41^ and anti-fibrotic in the latter^33^. This may be explained by tissue-specific differences (e.g. in cellular autophagy), however, these effects are yet to be confirmed in humans. We have shown that severe AS is independently associated with raised serum OPG, and that patients with chronic ischaemia pattern fibrosis on CMR have raised OPG on univariate analysis. In patients with infarction, this is due to presence of coronary artery disease, but also due to a lower mean ejection fraction and higher serum CRP. In this way, LV dysfunction, systemic inflammation, and CAD are likely to mediate serum OPG in patients with infarction on CMR, in addition to the degree of calcification of the aortic valve. The effect of the severity of AS on serum OPG, as determined by aortic valve area on CMR, was shown to be independent of age, sex, fibrosis on CMR, LV EF, and NYHA. This suggests that progressive calcification of the valve itself is the cause of the raise in serum OPG. Of course, this is likely to be affected by progression to pressure overload induced heart failure, or potentially the presence of concomitant cardiovascular disease, such as CAD, HTN, diabetes, which can be associated with systemic inflammation and/or endothelial dysfunction^16,42,43^. Indeed, the severity of endothelial dysfunction in these conditions is heterogeneous and directly affected by medications such as Angiotensin II receptor blockers (ARB) and ACE-inhibitors^44–46^, which were used in 46.3% of patients in our cohort, but prescription rates were similar between severity groups. Serum OPG may present a further prognostic marker for patients with severe AS, and may aid in determining the ideal timing for surgical intervention, but does not predict the presence of midwall fibrosis on CMR.

### Limitations

Our study was from a single centre and therefore subject referral bias, although we believe this was limited as our centre is a national referral centre with patients treated from across the country. In addition, our cohort contained predominantly Caucasian patients and therefore our results cannot be extrapolated to other ethnicities. Finally, we did not undertake T1-mapping with the CMR scanning, as it had not been validated in our institution at the time of recruiting our patients, and therefore we are unable to comment about the potential association between OPG and interstitial/diffuse fibrosis. Nonetheless, it is important to emphasise the predictive role of OPG in this cohort.

## Conclusion

There was no association between serum OPG and the presence of myocardial midwall fibrosis in our study. However, higher OPG levels were seen in patients with chronic myocardial infarction compared to patients with no fibrosis, which was due to increased levels of serum markers of inflammation, LV dysfunction, and CAD. We have confirmed the findings of others that OPG levels increase with AS severity, and that increased serum OPG levels are associated with poorer survival at mid-term follow-up (median of 1.9 years), independent of the presence of midwall fibrosis on the CMR. These data suggest that the RANKL/RANK/OPG axis is unlikely to play a central role in myocardial midwall fibrosis in patients with aortic stenosis.

## Electronic supplementary material

Supplementary table and figures

## Data Availability

Data can be obtained from the corresponding author and will be deposited in an open access repository upon acceptance.
