# Supplementary material for: Osteoprotegerin and Myocardial Fibrosis in Patients with Aortic Stenosis
Source: Sci Rep. 2018 Sep 28;8:14550. doi: 10.1038/s41598-018-32738-y (PMC6162228; doi:10.1038/s41598-018-32738-y)
Supplement: Supplementary file 1 — Supplementary table and figures [file 41598_2018_32738_MOESM1_ESM.pdf]

Osteoprotegerin and Myocardial Fibrosis in Patients with Aortic Stenosis

Brodie Loudon [a,b], Eleana Ntatsaki [c], Simon Newsome [a], Brian Halliday [a,d, #], Amrit Lota [a,d], Aamir Ali [a,d] , Tamir Malley [a], Subothini Selvendran [a,d], Nikhil Aggarwal [a,d], Willis Lam [a], Jackie Donovan [e], Dominque Auger [a], Claire E Raphael [a,d], Paul D Flynn [f], Dudley J Pennell [a,d], Vassilios S Vassiliou [a,b,d, #*], Sanjay K Prasad [a,d, *]

*Joint senior authors
# Joint corresponding authors

[a] CMR Unit, Royal Brompton Hospital and NIHR Biomedical Research Unit, Royal Brompton and Harefield Hospitals Imperial College London, London, United Kingdom
[b] Norwich Medical School, University of East Anglia, Bob Champion Research & Education Building and Norfolk and Norwich University Hospital, Norwich, United Kingdom
[c] Department of Rheumatology, Ipswich Hospital NHS Trust, and University College London, United Kingdom
[d] Imperial College London, London, United Kingdom
[e] Department of Biochemistry, Royal Brompton Hospital, London, United Kingdom
[f] Department of Metabolic Medicine, Addenbrooke's Hospital, Cambridge and University of Cambridge, United Kingdom

#Address for Correspondence:
Dr Brian Halliday
CMR Unit, Royal Brompton Hospital, Sydney Street,
London, SW3 6NP, UK
b.halliday@rbht.nhs.uk
Tel: +44 (0)20 7352 8121

Dr Vassilios Vassiliou
Norwich Medical School, University of East Anglia,
Norwich Research Park, United Kingdom NR4 7UQ, UK
V.Vassiliou@uea.ac.uk
Tel: +44 (0) 1603 592534

Supplementary Data

Table 1: Baseline Patient Data by Fibrosis Pattern on CMR

**Demographics**	**No Fibrosis (n=36)**	**Midwall Fibrosis (n=38)**	**Infarction Fibrosis (n=36)**	**P-Value**
Age, years	72 ± 12	77 ± 11	78 ± 7	0.025*
Male, n (%)	22 (61.1)	31 (81.6)	24 (66.7)	0.13
Hypertension, n (%)	20 (58.8)	17 (44.7)	20 (57.1)	0.44
SBP, mmHg	133 ± 19	130 ± 25	127 ± 18	0.49
DBP, mmHg	72 ± 15	72 ± 11	68 ± 10	0.25
Diabetes mellitus, n (%)	2 (6.9)	1 (3.0)	2 (6.7)	0.73
Current smoker, n (%)	3 (8.8)	1 (2.6)	1 (2.9)	0.52
Any coronary artery disease, n (%)	6 (16.7)	11 (28.9)	23 (63.9)	<0.0001*
Previous stroke, n (%)	0 (0.0)	3 (7.9)	0 (0.0)	0.11
Atrial Fibrillation, n (%)	4 (12.5)	2 (5.6)	5 (15.2)	0.44
Hypercholesterolaemia, n (%)	22 (66.7)	22 (59.5)	24 (68.6)	0.71
NYHA ≥ II	27 (75.0)	26 (68.4)	26 (72.2)	0.83
Height, cm	168 ± 12	172 ± 10	169 ± 10	0.26
Weight, kg	79 ± 20	82 ± 16	71 ± 15	0.026*
BMI, kg/m^2	28 ± 5	28 ± 4	25 ± 4	0.004*
**Medical therapy**				
Aspirin, n (%)	15 (44.1)	22 (59.5)	26 (76.5)	0.024*
Clopidogrel, n (%)	4 (12.1)	4 (11.1)	8 (25.0)	0.25
ACE I/ ARB, n (%)	14 (41.2)	13 (35.1)	24 (72.7)	0.004*
Beta Blocker, n (%)	11 (32.4)	17 (48.6)	18 (54.5)	0.16
Calcium channel blocker, n (%)	4 (12.5)	4 (11.8)	6 (18.2)	0.77
Diuretic, n (%)	11 (32.4)	23 (62.2)	23 (65.7)	0.010*
Warfarin, n (%)	5 (15.6)	3 (8.3)	2 (6.3)	0.49
Amiodarone, n (%)	3 (8.8)	1 (2.9)	0 (0.0)	0.27
Statin, n (%)	24 (70.6)	25 (67.6)	25 (73.5)	0.83
**Biochemical data**				
Osteoprotegerin , pmol/L	6.15 ± 2.73	7.15 ± 3.15	8.37 ± 4.16	0.023*
Creatinine, µmol/L	87 ± 21	100 ± 33	109 ± 41	0.065
**CMR data**				
CMR aortic valve area, cm^2	0.9 ± 0.4	0.9 ± 0.2	0.9 ± 0.2	0.81
Mild / Moderate AS, n (%)	13 (36.1)	11 (28.9)	11 (30.6)	0.82

Severe AS, n (%)	23 (63.9)	27 (71.1)	25 (69.4)	0.82
LVEF, %	66 ± 12	59 ± 18	51 ± 13	<0.0001*
LV Mass, g	153 ± 48	190 ± 66	158 ± 38	0.017*
LGE Mass	0 ± 0	7.0 ± 8.3	11.3 ± 7.2	-
LGE Percent	0 ± 0	3.7 ± 3.7	7.6 ± 5.9	-
**Intervention**				
None	20 (55.6)	14 (36.8)	11 (30.6)	0.13
AVR	11 (30.6)	11 (28.9)	12 (33.3)	0.13
TAVI	5 (13.9)	13 (34.2)	13 (36.1)	0.13

Values are mean ± SD unless otherwise stated. *p < 0.05. Baseline data for AS patients based on fibrosis pattern on CMR. ACE-I, angiotensin converting enzyme inhibitor; ARB, angiotensin II receptor blocker; AVR, aortic valve replacement; BMI, body mass index; CMR, cardiovascular magnetic resonance; DBP, diastolic blood pressure; LGE, late gadolinium enhancement; LVEF, left ventricular ejection fraction; NYHA, New York Heart Association; SBP, systolic blood pressure; TAVR, transcatheter aortic valve replacement.

Supplementary Figure 1

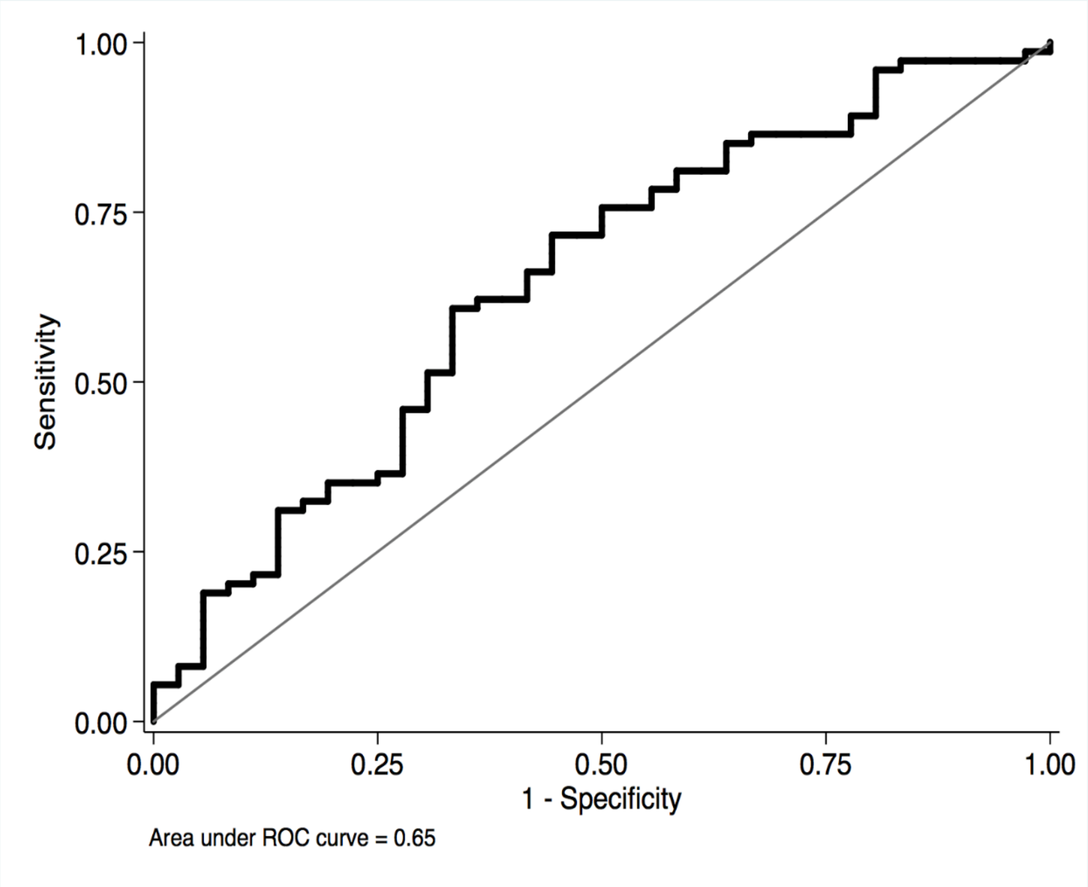

Receiver-operating characteristic (ROC) curve analysis for predicting AS severity. ROC curve describes the diagnostic performance of serum OPG in the distinction of severe and mild/moderate AS. Area under the ROC curve (AUC) was 0.69.

Supplementary Figure 2

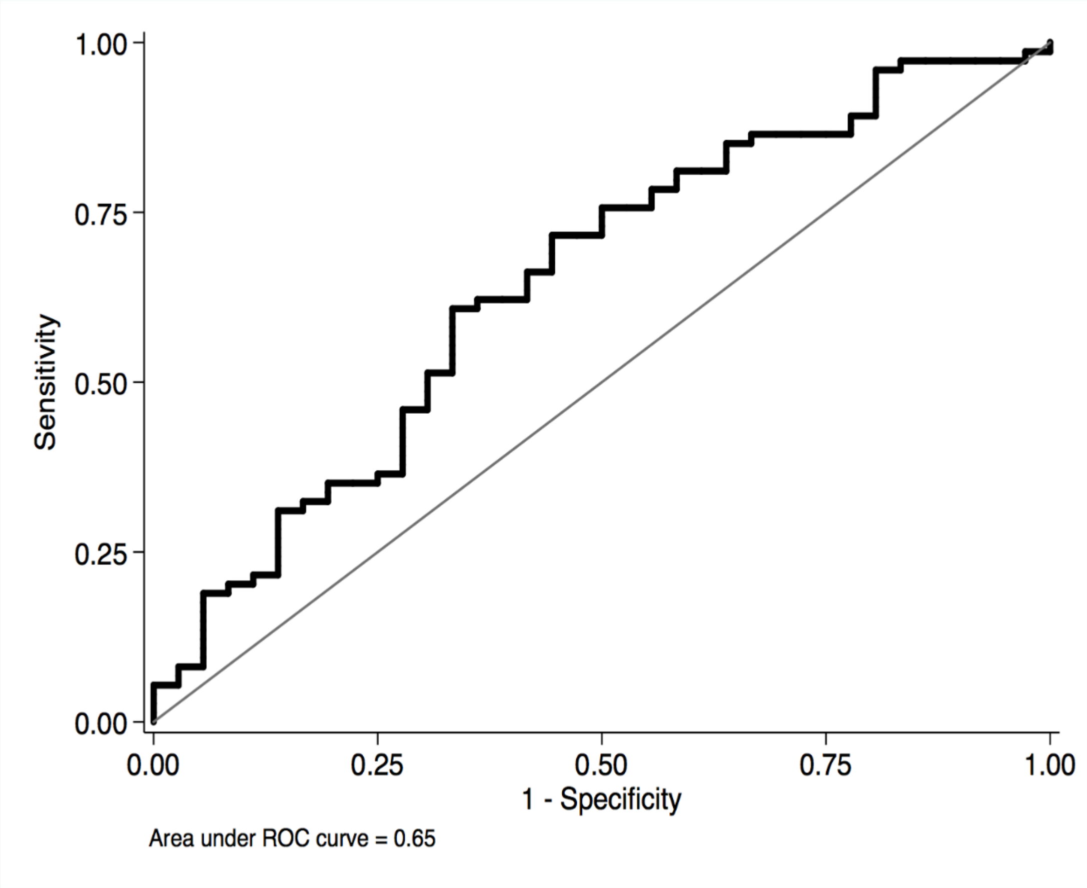

Receiver-operating characteristic (ROC) curve analysis for predicting LV fibrosis. ROC curve describes the diagnostic performance of serum OPG in the distinction of 'any fibrosis' and 'no fibrosis' on CMR. Area under the ROC curve (AUC) was 0.65.
